# The mitochondrial genome of *Lasioderma serricorne* (Coleoptera, Anobiidae)

**DOI:** 10.1080/23802359.2017.1422400

**Published:** 2018-01-03

**Authors:** Qiongyou Liu, Xiaohong Jiang, Xiaohui Hou, Renlian Cai, Jun Tan, Wenlong Chen

**Affiliations:** a Department of Basic Medical Sciences, Zunyi Medical University, Zunyi, People’s Republic of China;; b Guizhou Provincial Key Laboratory for Agricultural Pest Management of Mountainous Region, Special Key Laboratory for Development and Utilization of Insect Resources of Guizhou, Institute of Entomology, Guizhou University, Guiyang, People’s Republic of China

**Keywords:** Anobiidae, mitochondrial genome, *Lasioderma serricorne*

## Abstract

In this study, the complete mitochondrial genome of the *Lasioderma serricorne* was sequenced and analysed. The mitochondrial genome is 14,476 bp long and contains 13 protein-coding genes, two rRNA genes, 22 tRNA genes, and two non-coding region. Twenty three genes were found to be encoded by the majority strand and the other 14 genes by minority strand, those similar to that of other insects. The nucleotide compositing of the majority strand is 39.74% of A, 11.20% of C, 40.47% of T, and 10.39% of G. The phylogenetic analysis by maximum-likelihood (ML) method revealed that the *L. serricorne* was close to *Lasioderma redtenbacheri.*

The cigarette beetle, *Lasioderma serricorne* (Fabricius) (Coleoptera: Anobiidae) is a pest of durable grain commodities, spices, and stored tobacco. Larvae cause most feeding damage to commodities (Mahroof 2013). However, little information about its genetic characteristic has been reported. Therefore, we determined to sequence the complete mitochondrial genome of *L. serricorne* using the *De Novo* sequencing techniques strategy, with the purpose to studying biogeographic, molecular, and population studies. The adult *L. serricorne* was obtained from tobacco warehouses in Guiyang Tobacco Redrying Factory, Guizhou, China (GPS 26.52094N, 106.67497E), and then was reared on tobacco in lab. The voucher specimens are deposited in Institute of Entomology, Guizhou University, Guiyang, Guizhou, China (GZU-CO-000016). The fourth larvae stage of *L. serricorne* was washing with 70% ethanol first, and then those were stored in 95% ethanol, the mitochondrial DNA was extracted using *De Novo* sequencing library, and DNA sequencing at TGS (Total Genomics Solution Institute, Shenzhen, China).

The entire sequence of *L. serricorne* mitochondrial genome (GenBank accession no. MG592705) is 14,476 bp in length, consisting of 13 protein-coding genes, two ribosomal RNA (rRNA) genes, 22 transfer RNA (tRNA) genes, and two non-coding region (one A-T rich region and one control region). Twenty three genes (*trnI, trnM, nad2, trnW, cox1, trnL2, cox2, trnK, trnD, atp8, atp6, cox3, trnG, nad3, trnA, trnR, trnN, trnS1, trnE, trnT, nad6*, *cytB*, and *trnS2*) were found to be encoded by the majority strand (J-strand) and the other 14 genes (*trnQ, trnC, trnY, trnF, nad5, trnH, nad4, nad4L, trnP, nad1, trnL1*, *rrnL, trnV*, and *rrnS*) by minority strand (N-strand), those similar to that of other insects (Lin et al. 2017; Singh et al. 2017). Overall nucleotide compositions of the majority strand are 39.74% of A, 11.20% of C, 40.47% of T, and 10.39% of G, with an AT content of 80.21%.

The protein-coding genes begin with ATN start codon, such as *cox2*, *nad5*, and *nad1* start with ATA, *atp6*, *cox3*, and *nad4* genes employing ATG, while the rest using ATT as a start codon. Three types of stop codon are TAA (*cox2, atp6, cox3, nad2, cox1, nad4L*, and *nad6*), TAG (*nad5, nad1, atp8, nad3*, and *cytB*), and TTA (*nad4*). The *lrRNA* is located between *tRNAL1* and A-T rich region, whereas *srRNA* is accommodated between *tRNAV* and control region. The 22 tRNA genes vary from 61 to 71 bp in length. The secondary structure of tRNAs exhibited typical clover-leaf structure similar to other insect species. The size of the control region is only 136 bp (37.5% A, 5.15% G, 52.21% T, 5.15% C, with an AT content of 89.71%), and it located between *srRNA* and *tRNAI*, and the gene order around the control region is tRNAV-srRNA-control region-tRNAI-tRNAQ-tRNAM, and the order model was reported in some insects (Zhang and Hewitt 1997). Another noncodon region is an A-T-rich region, and it located between *tRNAV* and *lrRNA*.

The phylogenetic tree of *L. serricorne* was constructed with the 13 protein coding genes from 14 Coleoptera beetles by MEGA 7.0 (Kumar et al. 2016) using maximum-likelihood (ML) methods (Chen et al. 2016). As shown in Figure 1, the *L. serricorne* was close to *Lasioderma redtenbacheri.* Thus, this result supported the monophyly of *L. serricorne*.

**Figure 1.**
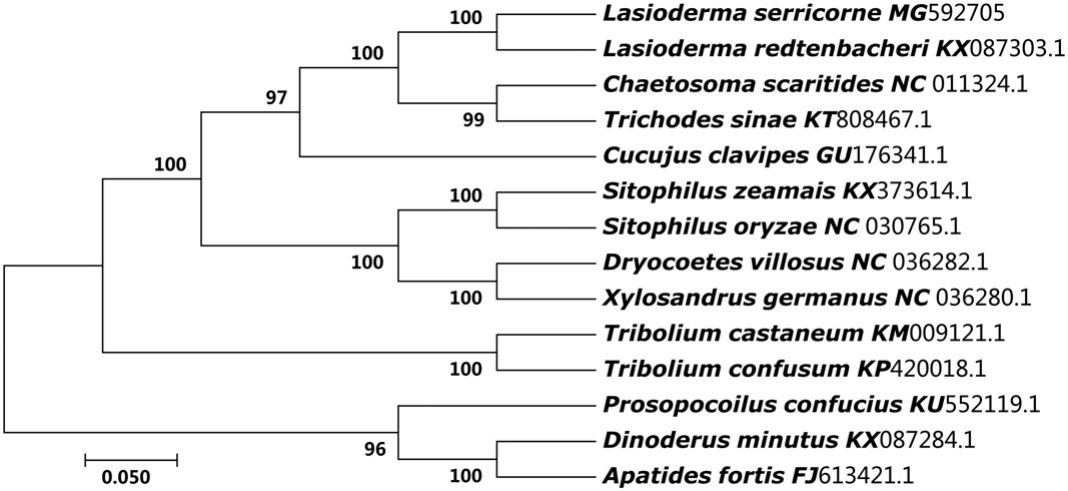
The ML phylogenetic tree of 14 beetles. The nucleotide sequences of the 13 protein-coding genes of each species in the complete or partial (only *Lasioderma redtenbacheri*) mitochondrial genome were downed from GenBank. The phylogenetic tree was constructed by MEGA 7.0 and Bootstrap support is shown at nodes.
